# Seaweed-based alginate/hydroxyapatite composite for the effective removal of bacteria, cyanobacteria, algae, and crystal violet from water

**DOI:** 10.1186/s13036-023-00387-z

**Published:** 2023-11-14

**Authors:** Mohamed Gomaa, Amal William Danial

**Affiliations:** Botany & Microbiology Department, Faculty of Science, Assiut University, Assiut, 71516 Egypt

**Keywords:** *E. coli*, Disinfection, Antialgal, Biosorption, Nanocomposite, nanohydroxyapatite

## Abstract

A novel cost-effective and multifunctional nanocomposite was developed based on sustainable macroalgae biomass. The brown seaweed *Sargassum latifolium* was utilized for alginate extraction and the calcareous red seaweed *Tricleocarpa fragilis* was utilized as CaCO_3_ source for nanohydroxyapatite synthesis. The developed Zn^2+^-crosslinked alginate/nanohydroxyapatite (ZA/nHA) beads were characterized by FT-IR, XRD, and TEM. The antimicrobial potential of ZA/nHA to disinfect synthetic *Escherichia coli*-contaminated water was evaluated at different bacterial load and composite concentrations. The developed ZA/nHA effectively inactivated bacteria at initial concentration ≤ 10^5^ CFU mL^−1^ and 0.5–1% (w/v) of ZA/nHA within 300–360 min. The kinetics of bacterial disinfection exhibited better fitting to Weibull model than Log-liner model, which confirmed the disinfection process. Furthermore, treatment of the cyanobacterium (*Chroococcus* sp.) and the microalga (*Chlorella* sp.) with ZA/nHA showed promising antialgal properties as indicated by reductions in chlorophyll a. The treatment indicated 100% and 90% removal of *Chroococcus* sp. and *Chlorella* sp. within 2 and 4 days, respectively. The developed ZA/nHA also exhibited a promising application as a biosorbent for crystal violet (CV). The adsorption process was very fast (0.171 mg CV g^−1^ adsorbent was removed within 7 min at pH 6.0). The adsorption kinetics exhibited better fitting to the pseudo-second order and Elovich models than the pseudo-first order equation. Besides, Sips model better represented the isotherm data of CV adsorption. The thermodynamic analysis indicated exothermic adsorption, which became more favorable at low temperature and high CV concentration. The developed nanocomposite is eco-friendly and suitable for multiple environmental applications.

**Graphical Abstract:**

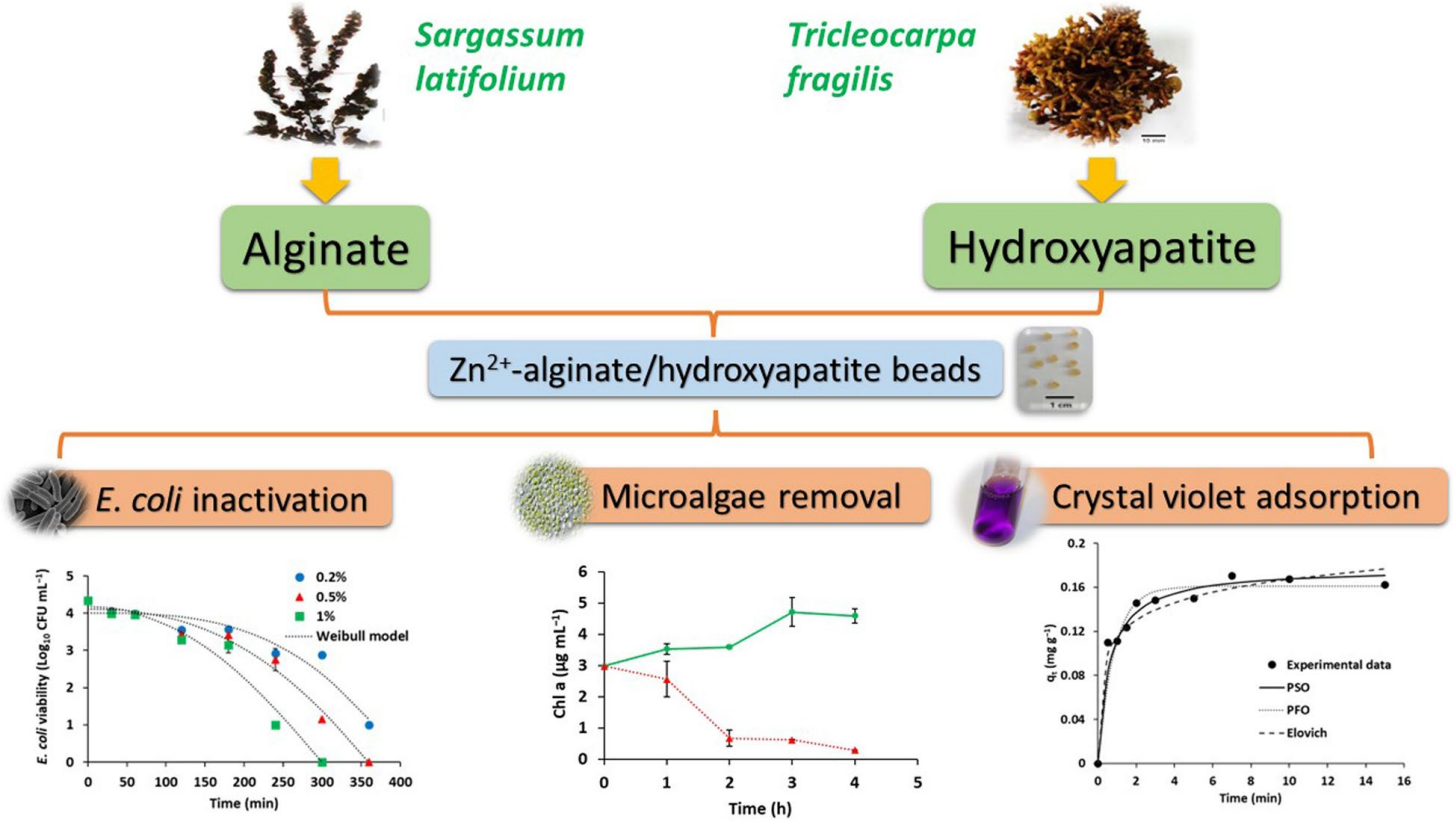

**Supplementary Information:**

The online version contains supplementary material available at 10.1186/s13036-023-00387-z.

## Introduction

Accessing clean, safe, and sustainable resources of water as well as the removal of hazardous contaminants from polluted water resources are one of the main demands of the current century. Microbial contamination of drinking water generally threatens human life by transmitting serious diseases such as diarrhea and dysentery [1]. The total number of coliforms and *Escherichia coli* in drinking water must be zero according to the guidelines of the Environmental Protection Agency [2] and the World Health Organization [1]. On the other hand, the increase of nutrients in the aquatic environment results in the development of harmful cyanobacterial and algal blooms, which also represent an enormous danger to human health and the whole ecosystem [3]. Similarly, aquatic pollution by persistent and toxic dyes from various industries can cause severe damage to ecosystem and life-threatening diseases after accumulation in human body [4]. Cystal violet (CV) is a common cationic dye in textile industry as well as in the preparation of paints and printing ink [5]. CV is also utilized as a biological stain, a skin disinfectant, and a bacteriostatic agent in the medical community and veterinary medicine. Besides, it is usually incorporated into poultry feed to prevent the growth of pathogenic microorganisms [6]. Despite its extensive applications, CV is carcinogenic and generally regarded as recalcitrant compound since it is non-biodegradable and can persist in different environments [5, 6].

In general, the common practices for the disinfection of water supplies include chemical methods (chlorine, ozone, iodine) and physical methods (UV radiation) [7]. Although these techniques are effective in eliminating undesired microbes, they can induce the formation of harmful byproducts that are carcinogenic [8]. Furthermore, the traditional practices for the treatment of dye-bearing wastewater include adsorption, membrane filtration, coagulation, ozonation, electrolysis, and photocatalysis as well as biodegradation using microorganisms and enzymes [9]. Among these technique, adsorption-based processes have been identified as a promising technology owing to their comparatively low operation costs, simplicity, and effectivity [10, 11].

Marine macroalgae are valuable and sustainable resources for the production of cost-effective materials for various applications. The seaweed biomass of Phaeophyceae (brown algae) contains important structural polysaccharides such as alginates. Alginate is the salt of alginic acid, which is a linear anionic hetero-polysaccharide consisting of D-mannuronic acid (M) and L-guluronic acid (G) [12]. The M and G residues occur in varying relative proportions and are organized in different blocks as MM, MG, and GG within the same chain of alginate [13]. Alginates are generally characterized by the ability to cross-link divalent cations such as Ca^2+^ and Zn^2+^, resulting in the formation of insoluble hydrogels with 3-D structures. Generally, alginates are exploited in different industries and biotechnological applications as thickeners, chelators, encapsulators and gel-forming agents [12].

On the other hand, some members of the Rhodophyceae (Red algae) are calcareous, which contain CaCO_3_ as a structural component in their skeleton. Although calcified macroalgae are widely distributed all over the world, their CaCO_3_ skeletons are less explored due to poor stability [14]. One route for the effective utilization of calcified biomass in biotechnology may rely on the conversion of hard CaCO_3_ skeletons into more stable material such as hydroxyapatite. Hydroxyapatite (HA) (Ca_10_(PO_4_)6(OH)_2_) is a promising bioactive ceramic material which is similar in composition and mechanical strength to the hard tissues of the human body [15]. However, little information is available in the literature regarding the utilization of calcified marine macroalgae for the preparation of nanohydroxyapatite. One study synthesized hydroxyapatite nanocrystals from mineralized *Corallina officinallis*, red alga, by thermal and chemical route [14]. However, no attempts have been carried out to develop nanohydroxyapatite from calcified red algae using chemical treatment only. The use of hydroxyapatite is generally limited by its brittleness and regeneration, which is avoided by formation of polymer/hydroxyapatite composite [16]. For instance, alginate/nanohydroxyapatite composites have gained a growing attention to combine the unique features of these compounds and produce hybrid materials with important characteristics such as biodegradability, non-toxicity, biocompatibility, etc. [17, 18]. Alginate/nanohydroxyapatite have been utilized as an eco-friendly composite for the adsorptive removal of methylene blue [16], lead [17], and fluoride [18]. Furthermore, this hybrid material exhibited excellent antibacterial properties against food-borne pathogens [19]. However, to the best of our knowledge, this is the first attempt of utilizing macroalgae waste as a cost-effective biomass for the preparation of Zn^2+^-crosslinked alginate/nanohydroxyapatite composite. Furthermore, a novel method was employed for obtaining nanohydroxyapatite from calcified macroalgae based on direct chemical precipitation.

The aim of the current study was to utilize the sustainable biomass of macroalgae for cost effective preparation of Zn^2+^-crosslinked alginate/nanohydroxyapatite composite beads. The developed nanocomposite was evaluated for mutable purposes including the disinfection of *E. coli*-contaminated water and removal of cyanobacteria (*Chroococcus* sp.) and microalgae (*Chlorella* sp.). Furthermore, the nanocomposite was evaluated as a biosorbent for the removal of crystal violet from aqueous solutions. The kinetics of *E.coli* disinfection as well as the kinetics, isotherms and thermodynamics of dye adsorption were also studied.

## Materials and methods

### Macroalgal biomass

The brown seaweed *Sargassum latifolium* (Turner) C. Agardh and the red seaweed *Tricleocarpa fragilis* (Linnaeus) Huisman & R.A. Townsend were collected from the Red Sea coast, Egypt. The algal biomass was sun dried and pulverized using a home blender.

### Extraction of alginate

The brown seaweed biomass was treated with a 3% w/v citric acid solution for 1 h at room temperature [11]. Acidic pretreatment removes fucoidan and the crosslinked cations with alginate and produces insoluble alginic acid within the seaweed biomass [11]. Then, the residual biomass was collected by filtration and subjected to alkaline treatment. The alkaline treatment involved the use of 2% w/v Na_2_CO_3_ at 40 °C for 2h. During this process the insoluble alginic acid is converted into soluble sodium alginate [11]. The extracted biomass is separated by filtration and sodium alginate in the filtrate was precipitated by double volume of ethanol. After 24 h at 4 ºC, the precipitated sodium alginate (SA) was collected by filtration and oven dried (60 ºC).

### Preparation of hydroxyapatite

The calcified red alga (*T. fragilis*) was treated with 1 M HCl solution for 10 min to convert CaCO_3_ in the cell wall into soluble CaCl_2_ in the solution according to the following reaction:

$$2\mathrm{ HCl }+\mathrm{ CaCO}_{3} \to \mathrm{ CaC}{\mathrm{l}}_{2} +\mathrm{ CO}_{2} + {\mathrm{H}}_{2}\mathrm{O}$$

The CaCl_2_ solution was collected by filtration and converted into calcium hydroxide according to the following reaction:

$${\mathrm{CaCl}}_{2} + 2\mathrm{NaOH }\to {\mathrm{Ca}\left(\mathrm{OH}\right)}_{2} + 2\mathrm{NaCl}$$

The precipitated calcium hydroxide was separated from the solution by centrifugation (3800 g, 10 min), oven dried and used for the synthesis of nanohydroxyapatite (nHA).

In a typical synthesis, 1 M of Ca(OH)_2_ suspension is reacted with 0.6 M phosphoric acid under continuous stirring and the pH of the mixture is controlled by NH_4_OH solution at pH 10 to avoid the formation of calcium deficient apatite [19]. The expected reaction is as follows.

$$10 {\mathrm{Ca}\left(\mathrm{OH}\right)}_{2} + {6\mathrm{H}}_{3}{\mathrm{PO}}_{4} \to + {{\mathrm{Ca}}_{10}\left({\mathrm{PO}}_{4}\right)}_{6}{\left(\mathrm{OH}\right)}_{2} (\mathrm{Hydroxyapatite})+ 18 {\mathrm{H}}_{2}\mathrm{O}$$

The precipitated hydroxyapatite was separated by centrifugation, oven dried and pulverized into a fine powder.

### Preparation of alginate/hydroxyapatite composite beads

An aqueous solution of SA (2% w/v) was prepared in distilled water and mixed with 1% w/v) of nHA powder. The composite mixture was homogenized by shaking at 500 rpm for 3 h. The zinc alginate/nanohydroxyapatite (ZA/nHA) beads were prepared by dropwise addition of the composite mixture into an aqueous solution of ZnCl_2_ (0.1 M, 100 mL) and left to cross-link for 1 h at room temperature. The collected ZA/nHA beads were washed several times with distilled water and oven dried (60 ºC).

### Characterization of the developed nanocomposite

The morphology and particle size of nHA and the structure of the ZA/nHA were investigated using transmission electron microscope (JEOL JEM-100CX II) at the Electron Microscopy Unit, Assiut University. Fourier transform-infrared (FT-IR) spectra of the developed materials were obtained in the 4000–400 cm^−1^ region using a Nicolet IS 10 FT-IR spectrophotometer. The X-ray diffraction (XRD) analyses were performed using an X-ray diffractometer (Shimadzu XD-3A). The point of zero charge (pH_pzc_) of the developed nanocomposite was determined as described in previous studies [20, 21].

### Bacterial disinfection of synthetic-bacteria contaminated water

The antibacterial activity of the ZA/nHA composite for water disinfection was evaluated against *Escherichia coli* (OQ569760), as a common water and wastewater contaminant. The bacterial strain was inoculated into nutrient broth and incubated at 37 ºC for 1–2 days. The cultivated cells were harvested by centrifugation (3800 g, 15 min) and resuspended in 0.9% (w/v) saline solution in sterile 100 mL Erlenmeyer flasks to give a final cell concentration of approximately 10^4^, 10^5^, and 10^6^ CFU mL^−1^ (CFU: colony forming unit). Then, the ZA/nHA beads were added into the bacterial suspension at 0.2, 0.5, and 1% w/v, and the mixture was incubated at room temperature with shaking at 100 rpm using a reciprocal shaker. At predetermined time interval, aliquots of the treated bacterial samples were withdrawn and plated into nutrient agar after proper dilution. The plates were incubated at 37 ºC in the dark for 24 h and the bacterial colonies were counted using a colony counter.

### Repeated use of the ZA/nHA composite beads for bacterial disinfection

ZA/nHA composite beads was used to disinfect an *E. coli* suspension (10^4^ CFU mL^−1^) for 6 h at room temperature and 100 rpm. Afterwards, the ZA/nHA was collected by filtration, washed thrice with distilled water followed by 70% v/v ethanol for 5 s, and oven dried at 60 ºC for 1 h. The collected ZA/nHA was used for the next cycle of the bacterial disinfection up to 7 cycles. After each cycle, aliquots of the bacterial suspension were diluted and plated on nutrient agar to check the viable residual cells.

### Modelling of bacterial inactivation

The curves of *E. coli* disinfection (Log_10_ CFU mL^−1^ *vs.* time) were fitted to two kinetic equations: Log-linear [22] and Weibull model [23]. The equations of these models are as follows:

Log-linear:$${{Log}_{10}(N}_{t})={{Log}_{10}(N}_{0})-{K}_{max}\frac{t}{Ln (10)}$$

Weibull model:${{Log}_{10}(N}_{t})={{Log}_{10}(N}_{0})-{\left(\frac{t}{\delta }\right)}^{P}$ where *K*_*max*_ is disinfection rate constant. *N*_*0*_ and *N*_*t*_ are the initial population of *E. coli* and the surviving population at time t. *δ* is a scale parameter reflecting the time required for 90% cell reduction (1 Log_10_) of *E. coli* population. *P* is a shape parameter related to the curve shape as convex (*P* > 1), concave (*P* < 1) or linear (*P* = 1).

The two models were solved using Geeraerd Inactivation Model Fitting Tool (GInaFiT, v1.6) [24]. The comparison between the models was performed by comparing the coefficient of determination (R^2^) and root mean sum of squared errors (RMSE).

### Cyanobacterial and algal inactivation using ZA/nHA composite

The cyanobacterial cells of *Chroococcus* sp. and the green microalga *Chlorella* sp. were cultivated in Bold’s Basal medium [25] under continuous illumination (48.4 µmol m^−2^s^−1^) at 25 ºC with shaking (150 rpm). Algal cells were diluted using fresh medium to obtain an initial optical density at 750 nm of 0.2, then treated using 1% (w/v) of ZA/nHA beads in batch cultures. Control treatments without the composite beads were performed similarly. The algal growth inhibition was monitored daily by measuring chlorophyll a (Chl. a) contents, which is a reliable indicator of cell viability during inactivation experiments [26]. A known aliquot of the microalgal suspension was withdrawn, and the cells were collected by centrifugation (3800 g, 15 min). The chlorophylls were extracted using 1 mL methanol at 60 ºC, and the samples were recentrifuged. The absorbance of the supernatant was measured using a spectrophotometer at 653 and 666 nm and Chl. a contents were calculated using the following equation [27]:

$$Chl. a \left(\mu g {mL}^{-1}\right)=15.65 {A}_{666}-7.34 {A}_{653}$$

### Adsorption of crystal violet using ZA/nHA composite

A known concentration of crystal violet (CV) was prepared in distilled water and mixed with a known amount of the ZA/nHA beads in 100 mL glass bottles. The initial pH of the CV solution was adjusted to 6.0 using diluted HCl or NaOH solutions. The adsorption experiment was maintained in the dark at 25 ºC in a shaking incubator (150 rpm). An aliquot of the treated CV solution was withdrawn at predetermined time interval to determine the residual CV concentration. The concentration of the CV was determined by measuring the absorbance of the solution at its maximum wavelength (690 nm) using a spectrophotometer. The amount of CV adsorbed (q_t_, mg g^−1^) at time t (min) was estimated using the following equation:

$${q}_{t}=\frac{\left({C}_{0}-{C}_{t}\right)V}{W}$$

where C_0_ and C_t_ are the initial concentration of CV, and its residual concentration at time t, respectively. V represents the volume of the CV solution (L), and W is the weight of the adsorbent (g).

The adsorption of CV using the developed composite was investigated at different initial CV concentrations (2.5, 5, 10, and 20 mg L^−1^), different temperatures (25, 35, and 45 ºC), and different pH values (3, 4, 5, 6, 7, and 8).

### Modeling of CV adsorption

The kinetics of the CV adsorption using ZA/nHA composite beads was simulated using the pseudo-first order (PFO), pseudo-second order (PSO), Elovich, and intra-particle diffusion (IPD) models using their non-linear equations.

PFO:$${q}_{t}={q}_{e}\left(1-{e}^{{-k}_{1}t}\right)$$

PSO:$${q}_{t}=\frac{{k}_{2}{q}_{e}^{2}t}{1+{k}_{2}{q}_{e}t}$$

Elovich: $${q}_{t}=\frac{1}{\beta }\mathrm{ln}\left(1+\alpha \beta t\right)$$

IPD: ${q}_{t}={k}_{i}{t}^{0.5}+{C}_{i}$ where q_t_ and q_e_ (mg g^−1^) is the amount of CV adsorbed at time t and equilibrium, respectively. k_1_ (min^−1^) and k_2_ (g mg min^−1^) are the rate constants for the PFO and PSO, respectively. α (mg g^−1^ min^−1^) and β (g mg^−1^) were related to the initial adsorption rate and the extent of surface coverage, respectively. K_i_ (mg g^−1^ min^−0.5^) is the rate constant of the intra-particle diffusion and C_i_ (mg g^−1^) is a constant reflecting the thickness of the boundary layer.

The adsorption process of CV was also investigated using different isotherm models viz*.* Langmuir, Freundlich, Sips, Dubinin-Radushkevich (D-R), and Temkin isotherms [28].

Langmuir: $${q}_{e}=\frac{{q}_{m}{K}_{L}{C}_{e}}{1+{K}_{L}{C}_{e}}$$

Freundlich: $${q}_{e}={K}_{F}{C}_{e}^{1/n}$$

Sips:$${q}_{e}=\frac{{q}_{m}{K}_{S}{C}_{e}^{1/{n}_{S}}}{1+{K}_{S}{C}_{e}^{1/{n}_{S}}}$$

D-R: $${q}_{e}={q}_{m}{e}^{-\beta {\varepsilon }^{2}}$$ and $$\varepsilon =RT\mathrm{ln}\left(1+\frac{1}{{C}_{e}}\right)$$

Temkin:${q}_{e}=\left(\frac{RT}{{b}_{T}}\right)\mathrm{ln}({A}_{T}{C}_{e})$ where q_m_ (mg g^−1^) is the maximum amount of CV adsorbed by the ZA/nHA adsorbent. K_L_ (L mg^−1^), K_F_ (mg g^−1^ (mg L^−1^)^−1/n^), K_S_ (mg L^−1^)^−1/n^, and A_T_ (L mg^−1^) are the adsorption constants of the Langmuir, Freundlich, Sips, and Temkin isotherms, respectively. n and n_S_ are dimensionless exponents of the Freundlich and Sips isotherms, respectively. T (K) is the absolute temperature, and R (8.314 J mol^−1^ K^−1^) is the universal gas constant. β (mol^2^ J^−2^) is a constant of adsorption energy, and ε (J mol^−1^) is the Polanyi potential. b_T_ (J mol^−1^) is a constant related to heat of adsorption.

Different kinetic and isotherm models were solved in Excel using Microsoft Solver function by minimizing the average relative error (%) (Eq. 1).

1 $$\%ARE= \frac{100}{N} \sum_{i=1}^{N}\left(\frac{\left|{q}_{pred}-{q}_{exp}\right|}{{q}_{pred}}\right)$$

where *q*_*pred*_ and *q*_*exp*_ refers to the predicted and the experimental amount of CV adsorbed, respectively. *N* represents the number of experimental points.

Additionally, temperature-dependent isotherms were used to investigate the thermodynamic process of CV adsorption based on the following equations [29]:

$${K}_{d}=\frac{{q}_{e}}{{C}_{e}}$$

$$\Delta G^\circ =-RT\mathrm{ln}{K}_{d}$$

$$\mathrm{ln}{K}_{d}=-\frac{\Delta H^\circ }{RT}+\frac{\Delta S^\circ }{R}$$

where K_d_ (L mg^−1^) is the distribution coefficient. ΔGº (J mol^−1^), ΔHº (J mol^−1^), and ΔSº (J mol^−1^ K^−1^) are the change of Gibbs free energy, enthalpy and entropy of the adsorption process, respectively. R (8.314 J mol^−1^ K^−1^) is the universal gas constant and T (K) is the absolute temperature. The plot (ln K_d_ *vs.* 1/T) was used to calculate the ΔHº and ΔSº by linear regression.

## Results and discussion

A novel cost-effective and sustainable Zn^2+^-crosslinked alginate/nanohydroxyapatite (ZA/nHA) composite was developed based on the brown macroalga *S. latifolium* as a source of alginate and the calcified red macroalga *T. fragilis* as a material for hydroxyapatite synthesis (Fig. S1). The extracted alginate had a viscosity average molecular weight of 1.76 × 10^5^ Da and mannuronic/guluronic acid ratio of 1.79. The pH_pzc_ of the developed ZA/nHA beads was 6.55.

### FT-IR analysis

**Fig. 1:**
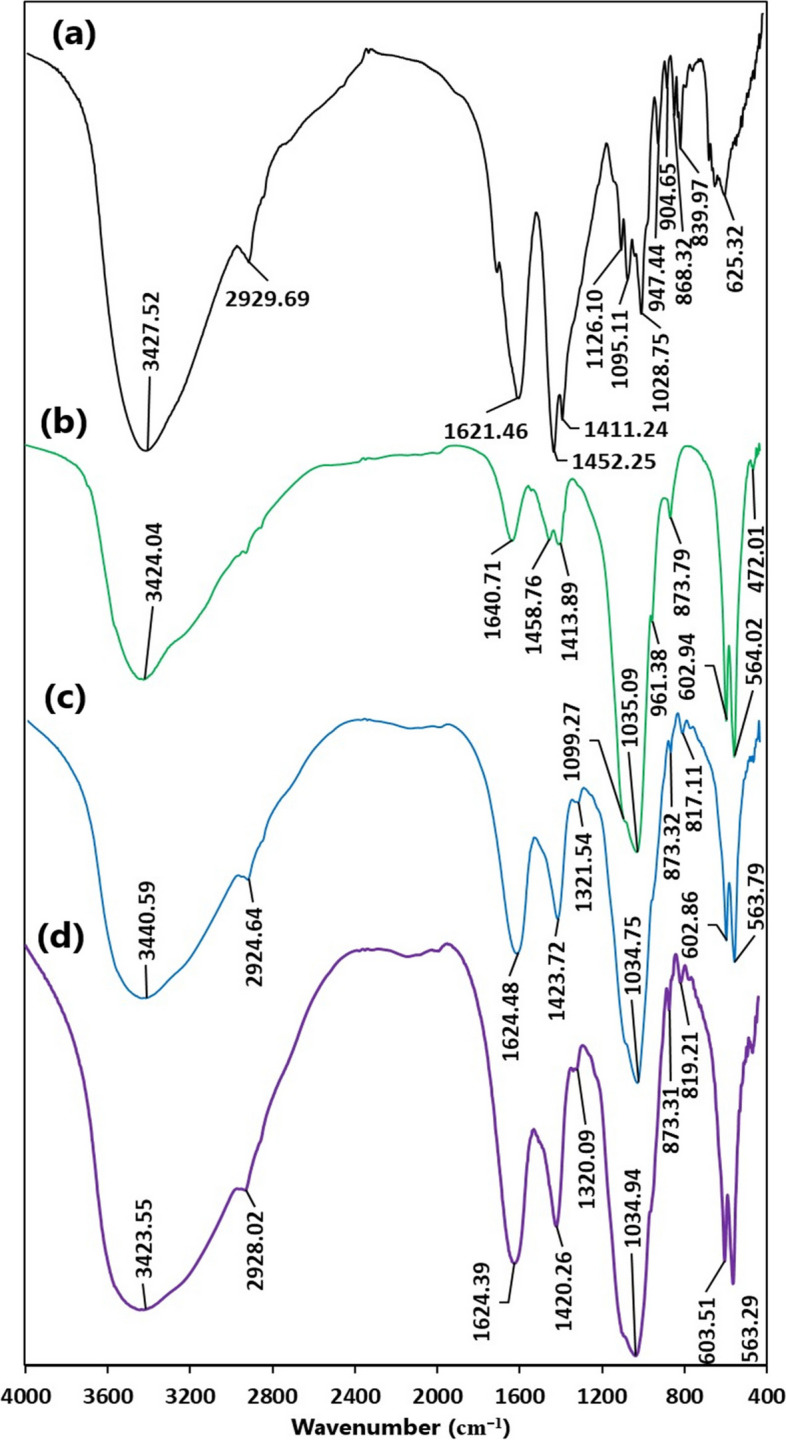
FT-IR spectra of (**a**) alginate from *S. latifolium*, (**b**) nanohydroxyapatite synthesized using *T. fragilis* biomass, (**c**) zinc alginate / nanohydroxyapatite composite, and (**d**) zinc alginate / nanohydroxyapatite composite after the adsorption of crystal violet

The hydrogen bonded O–H and C-H stretching vibrations in the macroalgal-derived alginate were observed at 3427.52 and 2929.69 cm^−1^, respectively (Fig. 1a). The sharp bands centered at 1621.46 and 1411.24 cm^−1^ were attributed to the asymmetric and symmetric − COO^−^ vibrations, respectively [30, 31]. While the C − C stretching vibration was located at 1452.25 cm^−1^. Furthermore, the stretching vibrations of C − O bonds were located at 1126.10 cm^−1^ and 1028.75 cm^−1^, while that at 1095.11 cm^−1^ indicated both C − O and C − C stretching vibrations [31]. The alginate spectrum exhibited a specific band of uronic acid at 947.44 cm^−1^ which was attributed to the C − O stretching vibrations (Fig. S1 b). Similarly, the bands at 904.65 and 839.97 cm^−1^ were assigned to the α-guluronic and β-mannuronic acid, respectively [31]. The band at 868.32 cm^−1^ was related to the C1-H deformation vibration of β-mannuronic acid [30].

On the other hand, the FT-IR spectrum of the phyco-genic nHA (Fig. 1b) indicated the existence of strong and sharp peak centered at 1035.09 cm^−1^ (ν_3_), and two weak peaks at 1099.27 cm^−1^ (ν_3_) and 961.38 cm^−1^ (ν_1_) were attributed to PO_4_^3−^ ions (stretching vibration of the P − O − P bond). The splitting sharp peaks at 602.94 and 564.02 cm^−1^ (ν_4_) reflected bending vibration of the PO_4_^3−^ ions, which occupied two sites in the crystal lattice [32]. Furthermore, the asymmetric stretching vibration of PO_4_^3−^ was indicated by a weak peak at 472.01 cm^−1^ (ν_2_) [33]. The stretching vibrations of the OH^−^ groups were assigned to the broad band at 3424.04 cm^−1^, with the band at 1640.71 cm^−1^ indicating the adsorbed or bounded H_2_O. Additionally, the presence of weak band at 873.79, 1413.89, and 1458.76 cm^−1^ may be assigned to CO_3_^2−^ anions substituting for the PO_4_^3−^ in the hydroxyapatite lattice (B-type hydroxyapatite) [34]. The carbonate that existed in the nHA was attributed to the adsorbed CO_2_ from the air, since the CaCO_3_ present in the seaweed biomass was solubilized using HCl during the first steps of nHA synthesis. Moreover, the existence of double sharp bands 564.02/602.94 and 1035.09/1099.27 cm^−1^ in the algal-derived n-HA reflected a high degree of crystallinity [33, 34].

The FT-IR spectrum of the ZA/nHA composite was depicted in Fig. 1c. New peaks located at 1624.48, 1423.72 and 1321.54 cm^−1^ were assigned to the COO^−^ groups of alginate and the one at 817.11 cm^−1^ may be identified as the combination of three possible vibrational modes (τ(CO) + δ(CCO) + δ(CCH)) of Zn-alginate [16]. Moreover, the shift in the COO^−^ groups in the composite compared to sodium alginate reflected the presence of electrostatic interactions and bond formation between the positively charged Ca^2+^ in the hydroxyapatite and COO^−^ groups of alginate [35]. In general, the FT-IR analysis confirmed a successful utilization of macroalgae wastes as cost-effective and sustainable approach for the fabrication of nanocomposite based on alginate and hydroxyapatite.

### XRD analysis

**Fig. 2:**
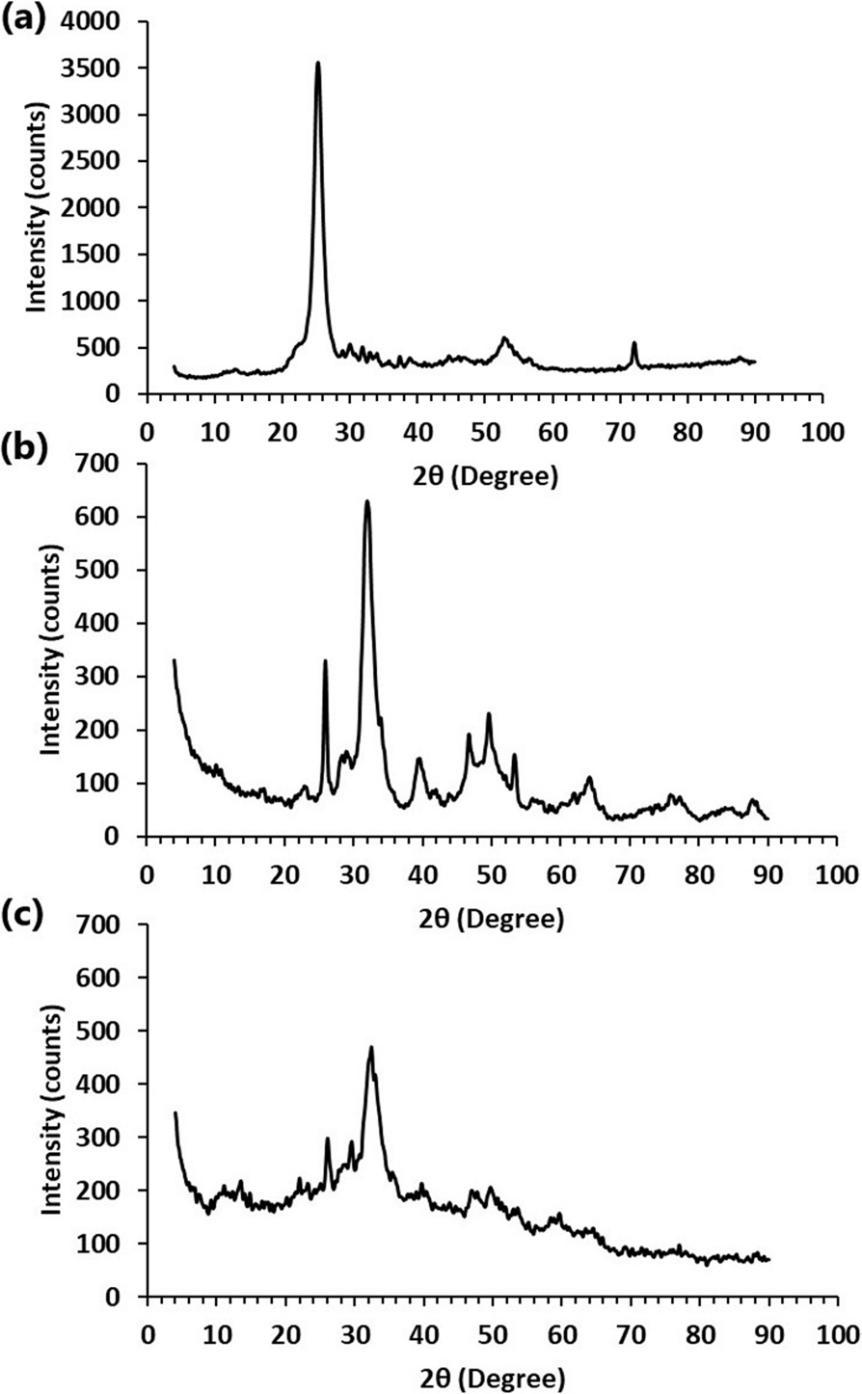
XRD spectra of (**a**) alginate, (**b**) nanohydroxyapatite, and (**c**) zinc alginate/nanohydroxyapatite composite

The XRD spectrum (Fig. 2a) of Na-alginate exhibited a distinctive peak between (2θ) 20° to 30°, which could be related to the amorphous nature of the polymer network [19, 36, 37]. The algal-derived nHA exhibited numerous peaks in the XRD spectrum (Fig. 2b), which were perfectly matched with standard card JCPDS: 01–074-0565 and indicates the formation of single phase of hydroxyapatite with the chemical formula Ca_10_(PO_4_)_6_(OH)_2_. Furthermore, the developed nHA showed hexagonal structure with a space group number 176 and a space group category P6_3_/m. The average crystallite size of nHA was 41.88 nm based on the Debye–Scherrer equation [16]. This size is quite similar to the nHA prepared from other natural sources such as bone [17], and egg shell [38].

On the other side, the spectrum of ZA/nHA beads exhibited characteristic peaks for both alginate and nHA (Fig. 2c). However, the spectrum of ZA/nHA indicated a shift and decrease in intensity of the major peaks for pure nHA and alginate, which implied the successful interaction between the nHA particles and alginate network.

### TEM analysis

**Fig. 3:**
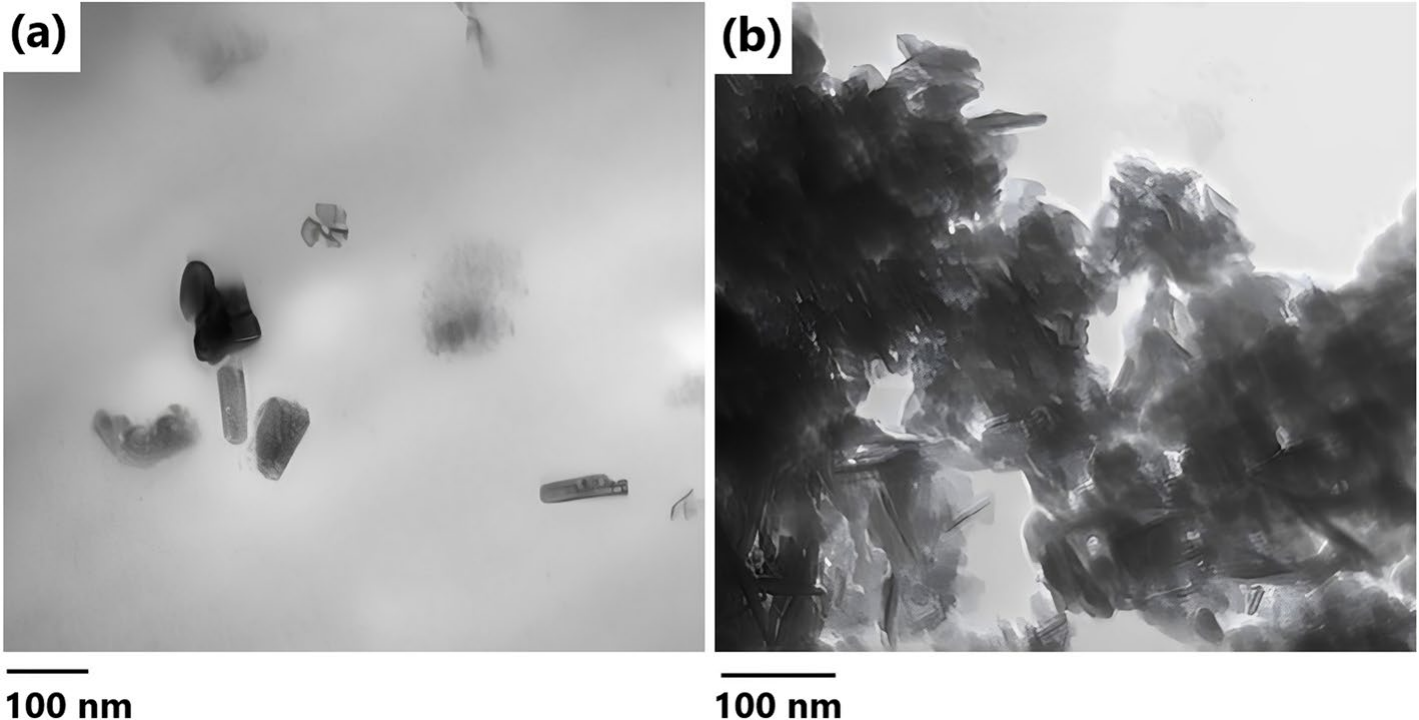
TEM images of (**a**) nanohydroxyapatite and (**b**) zinc alginate/ nanohydroxyapatite composite

Figure 3a depicts the TEM of the algal-derived hydroxyapatite. It was observed that the nHA particles had elongated ellipsoid-like shape. The crystallite size from TEM images using ImageJ software was 42.46 ± 17.92 nm. The TEM image of ZA/nHA beads showed the presence of agglomeration of nHA crystallites within the alginate matrix (Fig. 3b).

### Leaching of Zn^2+^ from the nanocomposite

The leaching of Zn^2+^ from the developed nanocomposite is of great importance, since it could influence its stability and application in water treatment as well as its environmental and health effects. As depicted in Fig. S2, the amount of Zn^2+^ released from the ZA/nHA composite was 1.50 and 2.16 mg L^−1^ after 6 and 24 h, respectively. The content of Zn^2+^ released remained relatively constant after 24 h. These values were generally lower than the maximum allowable concentration of 3 – 5 Zn^2+^ mg L^−1^ in drinking water suggested by WHO [1]. This result implied the safe use of the developed ZA/nHA composite for the treatment of water.

### Bacterial removal from contaminated water

**Fig. 4:**
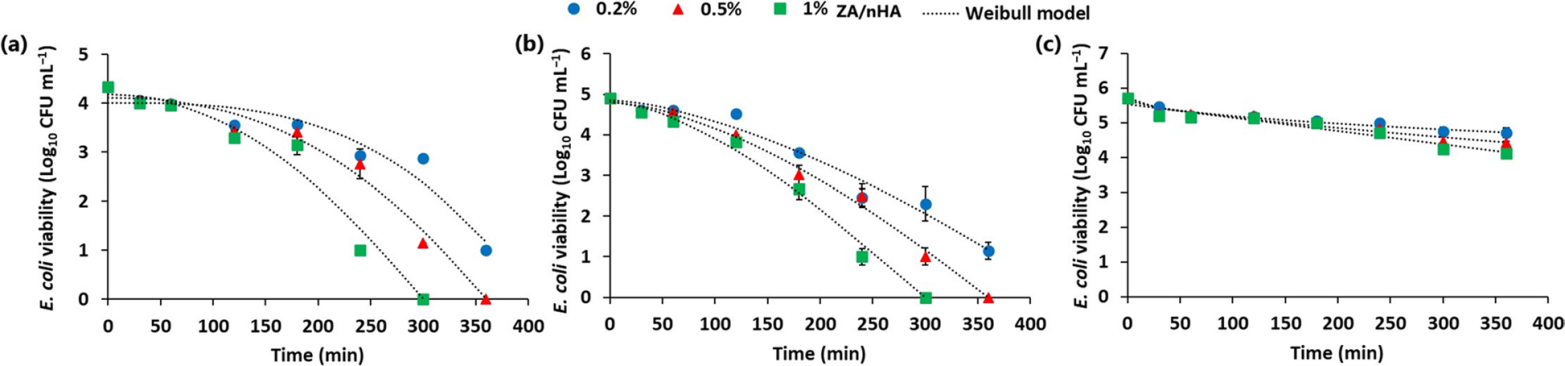
Kinetics of *E. coli* disinfection using different concentrations of zinc alginate/ nanohydroxyapatite beads at different initial bacterial concentrations of (**a**) 10^4^, (**b**) 10^5^, and (**c**) 10^6^ CFU mL.^-1^

The antibacterial activity of the developed ZA/nHA nanocomposite was tested against *E. coli*, a common pathogen in contaminated water (Fig. S3). The results depicted in Fig. 4 indicated that ZA/nHA exhibited concentration-dependent antibacterial activity. The effective disinfection of *E. coli* was related to the initial concentration of bacterial cells as well as the nanocomposite dosage.

The survival curves of *E. coli* in the presence of ZA/nHA nanocomposite were fitted to the Log-linear and the Weibull models (Table 1). Based on the R^2^ and RMSE values, the Weibull equation exhibited better fitting to the experimental results than the Log-linear equation. The inactivation kinetics of *E. coli* by the ZA/nHA are in good consistent with those of thermally-inactivated bacteria, which can also best described by the Weibull equation [39, 40]. The shape parameter (P) of the Weibull equation reflected downward concavity (*P* > 1), when the initial population of *E. coli* was ~ 10^4^ and 10^5^ CFU mL^−1^ (Table 1). Conversely, when the bacterial concentration was increased to ~ 10^6^ CFU mL^−1^, an upward concavity (*P* < 1) of the survival curve was evident except at 1% w/v of Zn/nHA, the curve was linear (*P* = 1) (Table 1). These empirical values could also reflect physiological effects of the nanocomposite on bacterial cells. At *P* > 1, the remaining cells of *E. coli* were increasingly damaged, while at *P* < 1, the cells may adapt to the applied stress [39]. These observations implied the effectiveness of the developed ZA/nHA composite in the disinfection of *E. coli*-contaminated water at populations ≤ 10^5^ CFU mL^−1^. Higher bacterial populations require prolonged treatment and/or higher composite dosage for effective disinfection.

**Table 1:** Kinetic modelling of bacterial disinfection under different treatments using Log-linear and Weibull models

**ZA/nHA (% w/v)**	**CFU mL**^**−1**^	**Log-linear model**			**Weibull modell**			
		**K**_**max**_	**R**^**2**^	**RMSE**	**δ**	**β**	**R**^**2**^	**RMSE**
0.2	10^4^	0.02	0.831	0.47	254.95	3.02	0.926	0.34
	10^5^	0.02	0.945	0.35	152.05	1.52	0.972	0.28
	10^6^	0.01	0.903	0.11	370.62	0.50	0.969	0.07
0.5	10^4^	0.03	0.892	0.55	195.13	2.33	0.981	0.25
	10^5^	0.03	0.964	0.37	131.86	1.57	0.993	0.18
	10^6^	0.01	0.929	0.13	282.03	0.73	0.937	0.13
1.0	10^4^	0.03	0.905	0.56	144.33	1.99	0.967	0.37
	10^5^	0.04	0.964	0.39	105.99	1.54	0.991	0.22
	10^6^	0.01	0.919	0.16	257.36	1.00	0.919	0.18

*K*_*max*_: disinfection rate constant. δ: scale parameter reflecting the time required for 90% cell reduction (1 Log_10_) of *E. coli* population. P is a shape parameter. R2: coefficient of determination. RMSE: root mean square error

### Cyanobacterial and algal removal from water

**Fig. 5:**
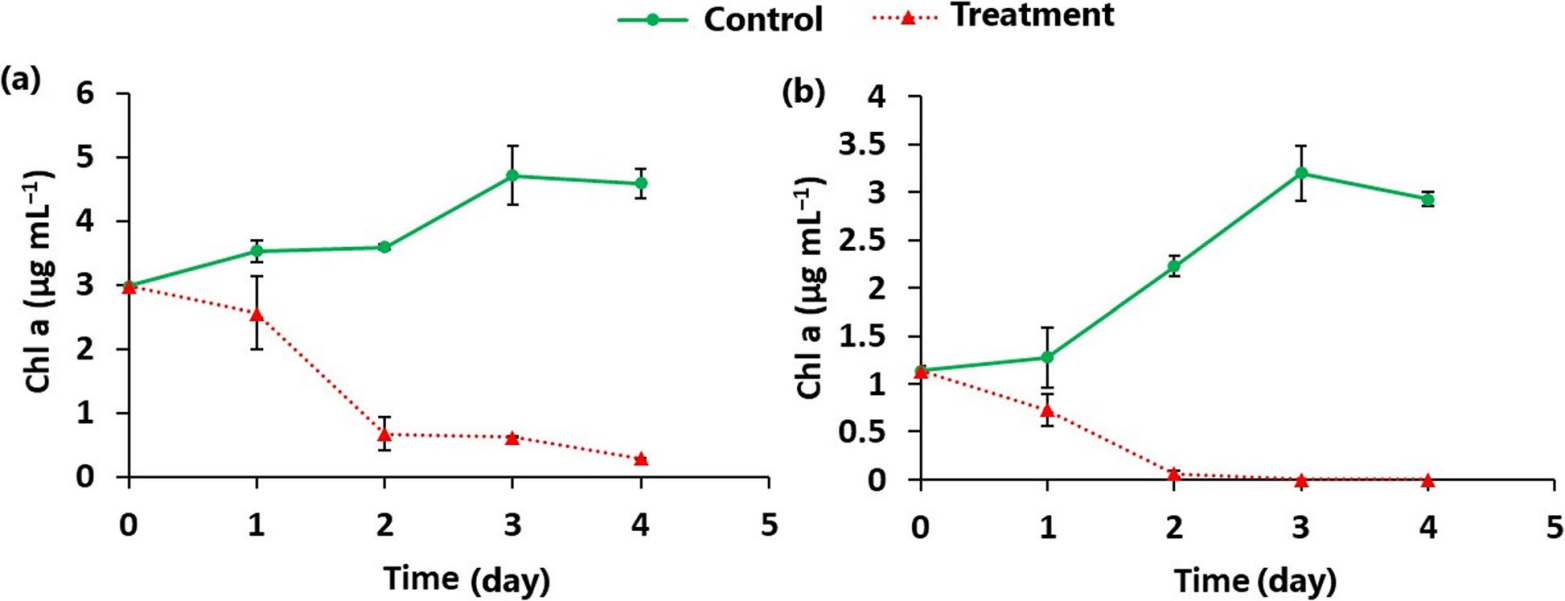
Time-dependent variations in Chl. a concentration of (**a**) *Chlorella* sp. and (**b**) *Chroococcus* sp. after treatment with 1% w/v of zinc alginate/ nanohydroxyapatite beads in relation to the untreated control

The effective removal of *Chroococcus* sp. and *Chlorella* sp. from water by ZA/nHA beads was monitored daily by measuring the concentration of Chl. a. The results depicted in Fig. 5 clearly demonstrated that ZA/nHA eliminated nearly 100% of *Chroococcus* sp. cells after 2 days of treatment. Conversely, the removal of *Chlorella* sp. reached ~ 90% after 4 days of treatment. More importantly, neither *Chroococcus* sp. nor *Chlorella* sp. was able to regrow after removing the beads and cultivation in a new medium.

### Adsorption of CV using ZA/nHA nanocomposite

**Fig. 6:**
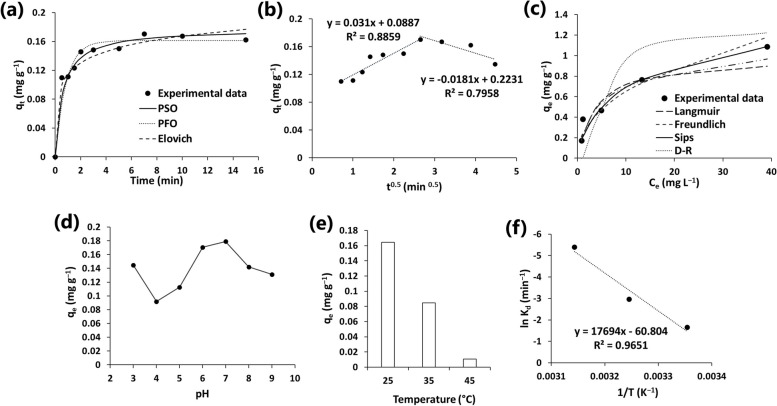
**a** Kinetics of crystal violet (CV) removal and fitting to pseudo-first order (PFO), pseudo-second order (PSO) and Elovich equations. **b** Kinetics modelling of CV adsorption using intra-particle diffusion model. **c** Effect of different initial CV concentrations on the adsorption process and fitting to different isotherm models. **d** Effect of pH on CV adsorption. **e** Effect of temperature on CV adsorption. **f** Thermodynamic plot for CV adsorption

The developed ZA/nHA nanocomposite exhibited fast adsorption properties towards CV (Fig. 6a). Accordingly, the nanocomposite adsorbed 0.11 mg g^−1^ of CV after 0.5 min and the maximum adsorbed amount was 0.17 mg g^−1^ at 7 min. However, a small degree of desorption was observed at prolonged contact time (Fig. 6a). Similarly, the adsorption of methylene blue using sodium alginate/hydroxyapatite composite was fast and the equilibrium was attained within short period (30 min) [16].

The kinetic mechanism of CV adsorption was evaluated using different kinetic equations and the results are listed in Table 2. The best fitting of the models was arbitrated based on high coefficient of determination (R^2^), and low error (%ARE). The results indicated that the PSO and Elovich equations better described the kinetics of CV adsorption on the surface of ZA/nHA than the PFO model (Fig. 6a, Table 2). The calculated equilibrium adsorption capacity (q_e_ = 0.178 mg g^−1^) by the PSO exhibited satisfactory fitting the experimental value (q_e_ = 0.171 mg g^−1^). The PSO model assumes that chemical adsorption is a rate-controlling mechanism for the removal of CV by ZA/nHA from aqueous solution. Similarly, Elovich model describes chemisorption and assumes that the adsorbent have heterogenous binding sites with various binding energies [41].

**Table 2:** Different parameters for the pseudo-first order (PFO), pseudo-second order (PSO), and Elovich models for the biosorption of crystal violet using zinc alginate/nanohydroxyapatite beads

**Models**	**Parameters**	
**Experimental**	***q***_***e***_ **(mg** **g**^**−1**^**)**	0.171
**PFO**	***K***_***1***_ **(min**^**−1**^**)**	1.18
	***q***_***e***_ **(mg** **g**^**−1**^**)**	0.161
	***R***^***2***^	0.780
	***%ARE***	9.36
**PSO**	**q**_**e**_ **(mg g**^**−1**^**)**	0.178
	**K**_**2**_**(g mg**^**−1**^ **min**^**−1**^**)**	9.50
	***R***^***2***^	0.878
	***%ARE***	5.60
**Elovich**	**β (g mg**^**−1**^**)**	43.08
	**α (mgg**^**−1**^ **min**^**−1**^**)**	3.16
	***R***^***2***^	0.868
	***%ARE***	5.15

The kinetics of CV removal were also fitted to the intra-particle diffusion model to determine the rate limiting step, and the results were depicted in Fig. 6b. The high R^2^ of the first linear region in the plot (q_t_ *vs* t^0.5^) deviated from the origin (Table 2). This implied that the intra-particle diffusion was prominent, but the deviation of the plot from the origin indicated a significant effect of the external mass transfer. The second linear region was related to the desorption of CV. The intra-particle rate constant (K_i_) was low in the second linear region, which reflected a slow desorption process in relation to the fast adsorption process.

The adsorption process of CV was also evaluated using different isotherms (Table 2, Fig. 6c). The investigated models exhibited satisfactory fitting to the experimental data, but the best fit was related to the Sips model, owing to relatively low %ARE values (Table 3). In general, the Sips model combines the assumptions of both Langmuir and Freundlich models [28]. The Langmuir model assumes the formation of a monolayer of the adsorbate molecules, which is chemically adsorbed at energetically homogenous sites [42]. However, the presence of different functional groups on the surface of ZA/nHA indicated their heterogenous behavior towards CV, which violates the basic principle of Langmuir model. Conversely, the assumption of the Freundlich model is related to the formation of multilayer of the adsorbate molecules at energetically different binding sites. At low pollutant concentrations, the Sips equation relatively reduces to the Freundlich model, but it can describe the monolayer coverage of the Langmuir model at high pollutant concentrations [28].

**Table 3:** Calculated parameter for different isotherm models for the adsorption of crystal violet using zinc alginate/nanohydroxyapatite beads

**Models**	**Parameters**	
**Langmuir**	**q**_**m**_ **(mg g**^**−1**^**)**	0.982
	**K**_**L**_ **(L mg**^**−1**^**)**	0.26
	***R***_***L***_	0.07 – 0.60
	***R***^***2***^	0.891
	***%ARE***	15.80
**Freundlich**	**N (mg g**^**−1**^**) (L mg**^**−1**^**)**^**1/n**^	2.25
	**K**_**F**_ **(mg g**^**−1**^**)**	0.23
	***R***^***2***^	0.960
	***%ARE***	14.62
**Sips**	**q**_**m**_ **(mg g**^**−1**^**)**	1.74
	***n***_***s***_	1.42
	**K**_**s**_ **(mg L**^**−1**^**)** ^**−1/n**^	0.13
	***R***^***2***^	0.959
	***%ARE***	10.02
**D-R**	**q**_**m**_ **(mg g**^**−1**^**)**	1.25
	***β*** (mol^2^ J^−2^)	4.57 × 10^-6^
	**E (kJ mol**^**−1**^**)**	0.33
	***R***^***2***^	0.892
	***%ARE***	50.58
**Temkin**	**A**_**T**_ **(L mg**^**−1**^**)**	2.89
	**b**_**T**_ **(kJ mol**^**−1**^**)**	12.11
	***R***^***2***^	0.935
	***%ARE***	13.69

An important parameter of the Langmuir isotherm is a dimensionless separation factor (R_L_), which is obtained as follows:

$${R}_{L}=\frac{1}{1+{K}_{L}{C}_{0}}$$

where K_L_ is the Langmuir constant and C_0_ is the initial CV concentration. This parameter signifies adsorption as unfavorable (R_L_ > 1), linear (R_L_ = 1), favorable (0 < R_L_ < 1), or irreversible (R_L_ = 0) [10]. The calculated R_L_ values in the present study fluctuated between 0.07 and 0.6 (Table 3), which implied favorable CV adsorption. In addition, the lower R_L_ values were observed at high CV concentration, which reflected that the adsorption process became more favorable at high CV concentrations. The dimensionless exponent of Freundlich model (n) can also be used to describe the nature of the adsorption as (*n* = 1), physical (*n* > 1), or chemical (*n* < 1) [41]. The n value for CV adsorption was 2.25 (Table 3), which demonstrated the heterogenous nature of the developed adsorbent and signified the adsorption of CV as favorable physical process. The existence of various binding sites with different binding energies was also supported by the satisfactory fitting of the Temkin isotherm. The b_T_ values of the Temkin model was 12.11 kJ mol^-1^, which is lower than 20 kJ mol^-1^, which is a characteristic feature of exothermic physical adsorption [43].

On the other side, the D-R isotherm showed the highest %ARE among the tested isotherms, suggesting a poor fitting to the experimental data. An essential property of the D-R isotherm is the calculation of mean free energy (E, kJ mol^-1^) of the adsorption using the following expression:

$$E=\frac{1}{\sqrt{2\beta }}$$

The E values signifies the adsorption process as chemical (E > 16 kJ mol^-1^), or physical (E < 8 kJ mol^-1^). The estimated E value for CV adsorption in the present study is 0.33 kJ mol^-1^ (Table 3), which indicated that the process is more inclined into physisorption.

On the other hand, pH of the solution during the adsorption process is a crucial parameter since it directly influences the surface charges of both adsorbent and adsorbate molecules. The effects of pH on the adsorption process of CV were depicted in Fig. 6d. The results indicated a maximum adsorption of CV at pH 6–7. The pH_pzc_ of the developed ZA/nHA beads was 6.55, which indicated its existence as a positively charged adsorbent at pH < pH_pzc_. Accordingly, increasing the pH enables the adsorption of ^−^OH ions on the surface of ZA/nHA beads, making it negatively charged and thus the removal of CV increased as a result of electrostatic interactions. However, the slight increase of CV adsorption at pH 3 may indicate that the adsorption is hydrogen-bonded controlled. The enhancement in the adsorption capacity of cationic dyes with the increasing pH agreed with previous studies [44].

Increasing temperature during adsorption markedly decreases the ability of the ZA/nHA to remove CV (Fig. 6e, f). The orientation and mechanism of CV adsorption was further investigated using thermodynamic parameters. The ΔHº value obtained in the current study for CV adsorption was negative, which is related to exothermic adsorption. The decrease of randomness at the adsorbent/adsorbate interface was indicated by the negative ΔSº value. Besides, the negative ΔSº suggested an associative adsorption mechanism without changes in the internal structure of the nanocomposite [29]. While the negative values of ΔGº signified the spontaneity of the thermodynamically favorable adsorption. The magnitude of ΔGº can also be used to identify adsorption mechanism as physisorption (ΔGº: − 20 to 0 kJ mol^-1^) or chemisorption (ΔGº: − 80 to − 400 kJ mol^-1^). Accordingly, the listed ΔGº values in Table S1 indicated that the adsorption of CV on the surface of ZA/nHA is physical, which agreed with the results of the D-R model. In a similar study, the adsorption of methylene blue was predominately physical on the surface of sodium alginate/hydroxyapatite material [16].

The FT-IR analysis of the nanocomposite beads after the adsorption of CV indicated a marked shift in the O–H stretching vibrations into lower wavenumber (3423.55 cm^−1^). Similarly, the peak of symmetric − COO^−^ vibrations was slightly shifted into lower wavenumber (1420.26 cm^−1^). Furthermore, A slight shift to the higher wavenumber was observed in the peaks of C-H (2928.09 cm^−1^), PO_4_^3−^ (603.51 cm^−1^), and the three vibrational modes of τ(CO) + δ(CCO) + δ(CCH) (819.21 cm^−1^). The shift in these functional groups in the nanocomposite implied their potential role in the removal of CV. The hydrogen-donating groups such as OH and COOH in the ZA/nHA adsorbent may form H-bonding interactions with the hydrogen accepting group (nitrogen) of CV. Furthermore, another type of H-bonding may occur between the H-donating groups of the adsorbent and the aromatic rings of CV, which is known as Yoshida bonding [10, 11]. Similarly, the presence of electron- donating oxygen groups in the ZA/nHA may induce the formation of n-π interaction with aromatic rings of CV as electron-acceptor. Furthermore, the electrostatic interactions were also evident as indicated by the effects of pH on the removal process.

## Conclusion

A novel cost-effective multi-functional composite was developed using alginate from the brown seaweed *S. latifolium* and hydroxyapatite synthesized by chemical precipitation using the red seaweed *T. fragilis* as CaCO_3_ source. The developed ZA/nHA was effectively applied to disinfect *E. coli* in water and the efficiency was increased by increasing the composite concentration and decreasing bacterial load. The kinetics of bacterial disinfection exhibited good fitting to the Weibull equation. Furthermore, the ZA/nHA showed potential application in the removal of cyanobacterial cells and microalgae after 2–3 days of treatment. On the other side, the developed composite showed fast adsorption removal of CV from the solution with q_e_ value equal to 0.171 mg g^−1^ within 7 min. The adsorption process was maximized at pH 6–7 which is suitable for the treatment of dye-polluted water without pH adjustment. The PSO and Elovich equations exhibited better fitting to the kinetics of CV adsorption than the PFO model. While the isotherm analysis indicated better fitting of the Sips model than Langmuir and Freundlich equations. The adsorption process was more inclined into physiosorption through H-bonding, Yoshida-boding, and n-π interactions and became more favorable at high concentration of CV. The results of the present study highlighted the promising application of sustainable macroalgae biomass for the development of cost-effective nanocomposite for the treatment of both drinking water and wastewater.

### Supplementary Information

**Additional file 1. **

## Data Availability

The datasets used and/or analyzed during the current study are available from the corresponding author on reasonable request.
