# Supplementary material for: Seaweed-based alginate/hydroxyapatite composite for the effective removal of bacteria, cyanobacteria, algae, and crystal violet from water
Source: J Biol Eng. 2023 Nov 14;17:69. doi: 10.1186/s13036-023-00387-z (PMC10644496; doi:10.1186/s13036-023-00387-z)
Supplement: Supplementary file 1 — Additional file 1. [file 13036_2023_387_MOESM1_ESM.docx]

**SUPPORTING INFORMATION**

**Seaweed-based alginate/hydroxyapatite composite for the effective removal of bacteria, cyanobacteria, algae, and crystal violet from water**

**Mohamed Gomaa*, Amal William Danial**

**Botany & Microbiology Department, Faculty of Science, Assiut University, 71516, Assiut, Egypt**

*** Corresponding author**

**Dr. Mohamed Gomaa**

**Botany and Microbiology Department, Faculty of Science, Assiut University, 71516 Assiut, Egypt**

**e-mail: m_gomaa@aun.edu.eg**

[**https://orcid.org/0000-0003-1544-3042**](https://orcid.org/0000-0003-1544-3042)


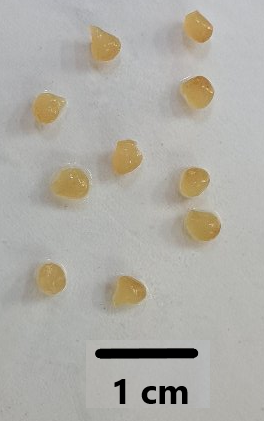


**Fig. S1:** Image of the developed zinc alginate/ nanohydroxyapatite beads after preparation.


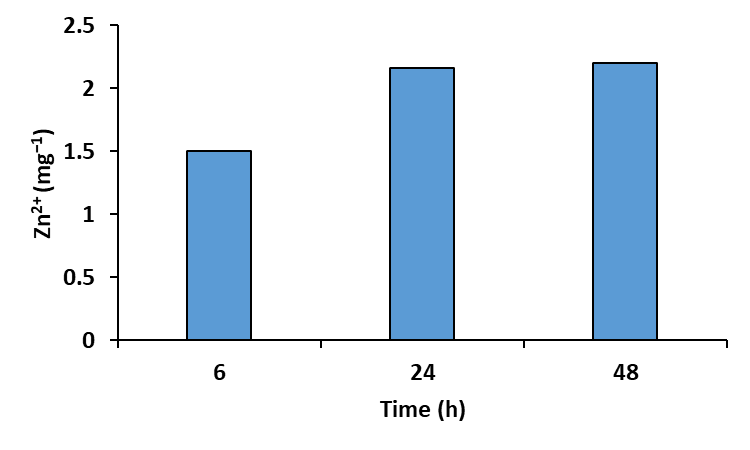


**Fig. S2:** Amount of zinc ions released in water from zinc alginate/ nanohydroxyapatite beads at different periods.


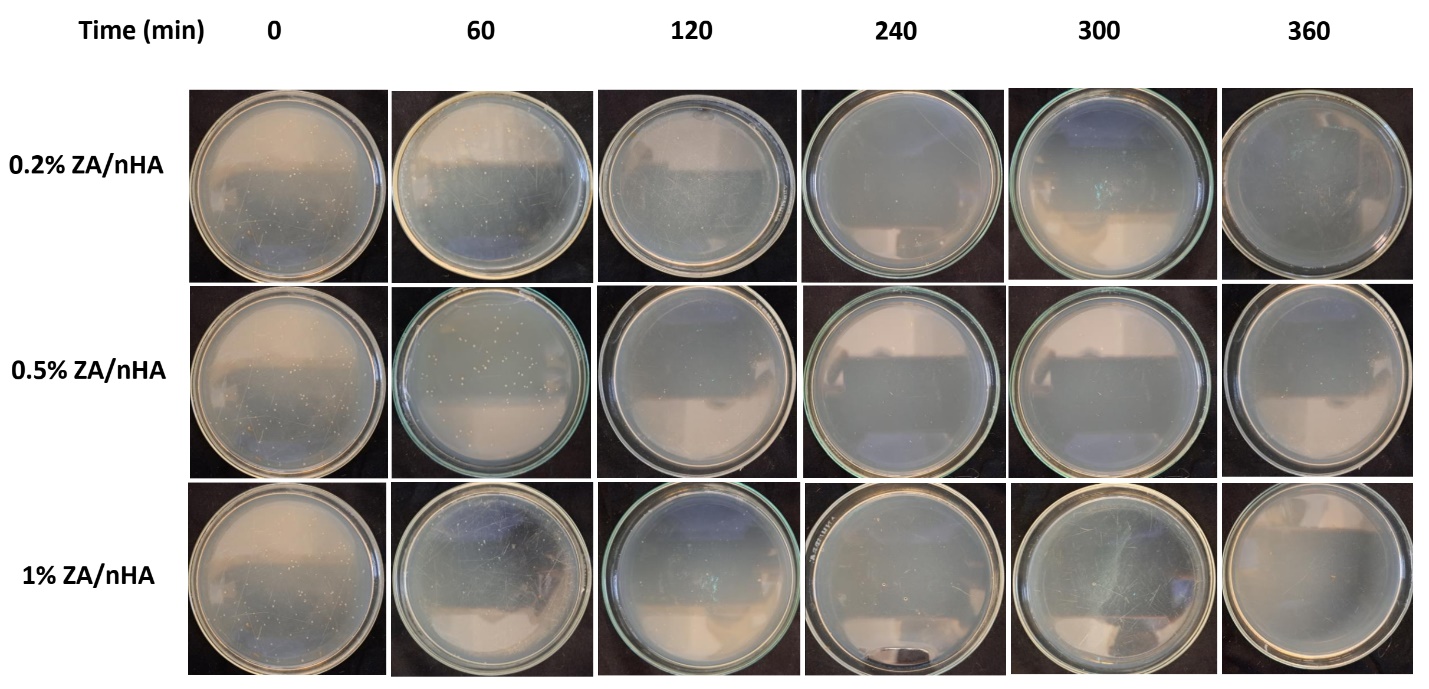


**(a)**


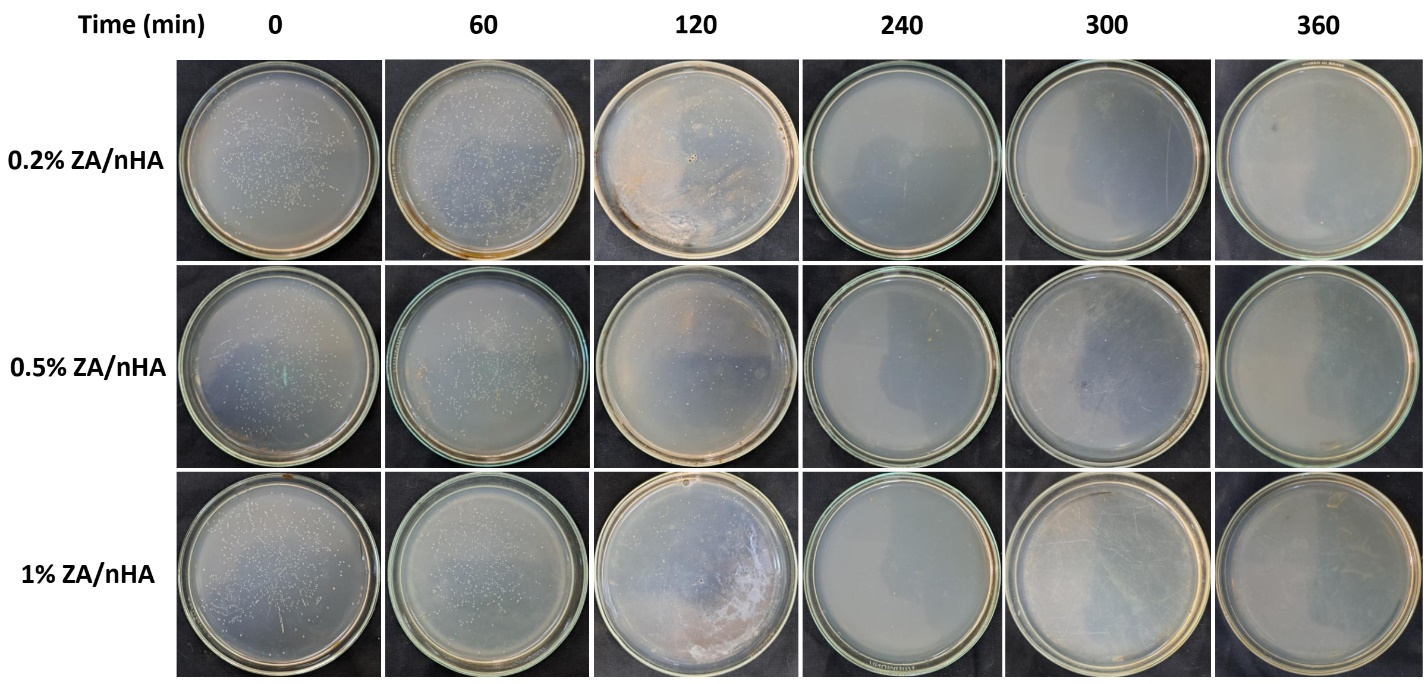


**(b)**


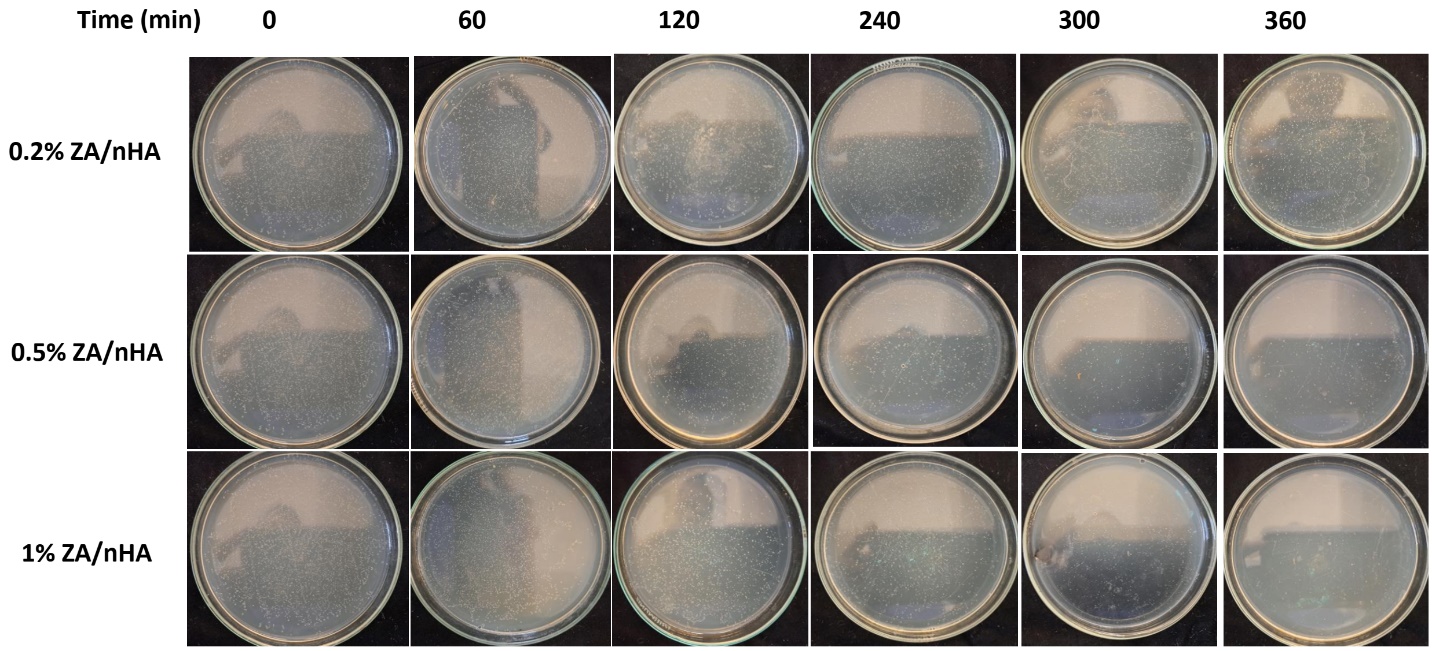


**(c)**

**Fig. S3:** Images of plates showing bacterial colonies remaining after different treatments with zinc alginate/ nanohydroxyapatite beads (ZA/nHA) at different initial bacterial concentration of: (a) 10^4^, (b) 10^5^, and (c) 10^6^ CFU mL^−1^.

| **Temperature (k)** | **ΔG (kJ mol^−1^)** | **ΔH (kJ mol^−1^)** | **ΔS (kJ mol^−1^)** |
| --- | --- | --- | --- |
| 298.15 | -0.47 | -147.12 | -0.51 |
| 308.15 | -0.13 |  |  |
| 318.15 | -0.12 |  |  |

**Table S1:** Thermodynamic parameters of crystal violet adsorption using ZA/nHA composite.
